# Photosynthetic capacity and assimilate transport of the lower canopy influence maize yield under high planting density

**DOI:** 10.1093/plphys/kiae204

**Published:** 2024-04-09

**Authors:** Yanyan Yan, Fengying Duan, Xia Li, Rulang Zhao, Peng Hou, Ming Zhao, Shaokun Li, Yonghong Wang, Tingbo Dai, Wenbin Zhou

**Affiliations:** Institute of Crop Sciences, Chinese Academy of Agricultural Sciences, Beijing 100081, China; Key Laboratory of Crop Physiology Ecology and Production Management, Ministry of Agriculture, Nanjing Agricultural University, Nanjing 210095, China; Institute of Crop Sciences, Chinese Academy of Agricultural Sciences, Beijing 100081, China; State Key Laboratory of Crop Gene Resources and Breeding, Institute of Crop Sciences, Chinese Academy of Agricultural Sciences, Beijing 100081, China; Institute of Crop Sciences, Chinese Academy of Agricultural Sciences, Beijing 100081, China; State Key Laboratory of Crop Gene Resources and Breeding, Institute of Crop Sciences, Chinese Academy of Agricultural Sciences, Beijing 100081, China; Ningxia Academy of Agriculture and Forestry Sciences, Crops Research Institute, Yinchuan 750105, China; Institute of Crop Sciences, Chinese Academy of Agricultural Sciences, Beijing 100081, China; Institute of Crop Sciences, Chinese Academy of Agricultural Sciences, Beijing 100081, China; Institute of Crop Sciences, Chinese Academy of Agricultural Sciences, Beijing 100081, China; Ningxia Academy of Agriculture and Forestry Sciences, Crops Research Institute, Yinchuan 750105, China; Key Laboratory of Crop Physiology Ecology and Production Management, Ministry of Agriculture, Nanjing Agricultural University, Nanjing 210095, China; Institute of Crop Sciences, Chinese Academy of Agricultural Sciences, Beijing 100081, China; State Key Laboratory of Crop Gene Resources and Breeding, Institute of Crop Sciences, Chinese Academy of Agricultural Sciences, Beijing 100081, China

## Abstract

Photosynthesis is a major trait of interest for the development of high-yield crop plants. However, little is known about the effects of high-density planting on photosynthetic responses at the whole-canopy level. Using the high-yielding maize (*Zea mays* L.) cultivars “LY66,” “MC670,” and “JK968,” we conducted a 2-yr field experiment to assess ear development in addition to leaf characteristics and photosynthetic parameters in each canopy layer at 4 planting densities. Increased planting density promoted high grain yield and population-scale biomass accumulation despite reduced per-plant productivity. MC670 had the strongest adaptability to high-density planting conditions. A physiological analysis showed that increased planting density primarily led to decreases in the single-leaf area above the ear for LY66 and MC670 and below the ear for JK968. Furthermore, high planting density decreased chlorophyll content and the photosynthetic rate due to decreased canopy transmission, leading to severe decreases in single-plant biomass accumulation in the lower canopy. Moreover, increased planting density improved presilking biomass transfer, especially in the lower canopy. The yield showed significant positive relationships with photosynthesis and biomass in the lower canopy, demonstrating the important contributions of these leaves to grain yield under dense planting conditions. Increased planting density led to retarded ear development as a consequence of reduced glucose and fructose contents in the ears, indicating reductions in sugar transport that were associated with limited sink organ development, reduced kernel number, and yield loss. Overall, these findings highlighted the photosynthetic capacities of the lower canopy as promising targets for improving maize yield under dense planting conditions.

## Teaser

Photosynthetic capacity and assimilate transfer of the canopy layer below the ear greatly contribute to maize grain yield under high planting density conditions in the field.

## Introduction

To meet the nutritional demands of the 9 billion humans who are predicted to live on earth by 2050, a 60% to 100% increase in crop production is necessary (Prajal et al. 2015; Tian et al. 2021). Target species for increased production include grains such as maize (*Zea mays* L.), which is a staple food throughout the world and is currently the most abundantly produced of all cereal crops (FAO 2021). As the second-largest maize producer, China contributes 23% of the global maize supply and contains 21% of the maize-growing area (FAO 2021). However, rapid urbanization, economic growth, and growing domestic meat consumption over the past 2 decades have led to a widespread increase in the occupation and fragmentation of arable land, including maize-growing land (Xin et al. 2023; Zhang et al. 2023). In the face of limited available arable land for maize growth, it is crucial to improve maize production per unit area to ensure food security.

Planting density is one of the most important agronomic practices in maize production (Zheng et al. 2017; Luo et al. 2023). Increasing planting density has been shown to improve maize yield by an average of 17% to 20% (Assefa et al. 2018). This approach can allow optimal use of available sunlight, promoting efficient conversion of carbon dioxide and water into maize grains (Simkin et al. 2019; Hu et al. 2020; Ma et al. 2020). However, dense planting can lead to intraspecific competition for available resources, namely light (in the aerial tissues) and nutrients and water (in the roots) (Deng et al. 2012; Duan et al. 2023). This can result in decreased per-plant growth and yield (Yan et al. 2021), although the effects vary significantly between maize varieties.

At the whole-field scale, vertical light interception and light absorption in specific canopy layers are strongly affected by the canopy architecture (Sarlikioti et al. 2011; Sultana et al. 2023). Previous studies have shown that variability in the light environment along the vertical canopy profile significantly impacts leaf physiology, energy dissipation, and photosynthetic capacity (Andrea et al. 2016). Therefore, photoassimilation (and subsequently yield) in each layer along the canopy profile is directly dependent on canopy architecture. Several studies have indicated that intermediate or upper leaves in the canopy contribute a lion’s share of maize carbon accumulation and grain yield (Allison and Watson 1966; Slattery et al. 2018; Xu et al. 2021). However, overall canopy productivity is still significantly affected by the lower layers. Increased planting density reduces light penetration into these layers (Timlin et al. 2014); plant shade responses thus strongly influence yield production under high planting density conditions. Decreased light availability can accelerate senescence and decreases radiation utilization efficiency, thus reducing per-plant yield (Zhang et al. 2019; Guo et al. 2021). Despite these prior findings, there is a still a lack of a systematic understanding of the relationship between canopy structure and maize grain yield.

Maize is a cross-pollination crop with 2 distinct inflorescences, referred to as the tassel (male) and the ear (female). These structures share common developmental processes in their early stages but have unique structural features at maturity that directly affect yield (Parvathaneni et al. 2020). Maize genotype is the primary determinant controlling ear and tassel development (Wilson and Allison 1978). However, crop management strategies and environmental factors, such as planting density, drought, shading, and soil fertility, also lead to variations in ear/tassel differentiation (Zhang et al. 2009; Hu et al. 2022). Previous studies have indicated that intraspecific competition for nutrients, water, and light can severely affect kernel number per ear and tassel size in maize planted at high density (Pagano et al. 2007; Zhang et al. 2018). Additionally, the extended anthesis–silking interval (ASI) induced by high density causes asynchronous flowering, hindering successful pollination and leading to yield losses of ∼40% to 50% (Uribelarrea et al. 2008; Sher et al. 2017). Thus, ASI is a critical trait contributing to density tolerance in maize, although the physiological mechanism underlying ASI-associated yield loss under high-density planting is largely unclear.

To delineate the mechanisms associated with maize single-plant yield loss under high-density planting conditions, we conducted a 2-yr field experiment in the high-yield maize ecosystem of northwestern China. Systematic analyses were carried out to characterize physiological changes in 3 high-yielding maize varieties grown at 4 planting densities. The response patterns of photosynthetic- and yield-related traits along the canopy layers were measured, and differences in the effects of planting density on ear and tassel development were assessed. This approach was designed to comprehensively reveal the regulatory mechanism(s) underlying planting density responses in 3 maize varieties, providing key insights into density tolerance traits to ultimately promote high-yield maize breeding.

## Results

### Effects of planting density on maize yield and population-scale biomass accumulation in the field

The 2-yr field experiments were conducted in Ningxia, China, during the growing seasons of 2019 and 2020 (Supplementary Fig. S1) to test the effects of planting density on yield (Table 1). Three maize varieties were planted at 4 densities: 75,000, 105,000, 120,000, and 135,000 plants/ha (D1 to D4, respectively), and aerial plant tissues were vertically divided into 4 layers (Layers I to IV) based on the ear position for canopy profiling (Supplementary Fig. S2). Population-scale maize yield was significantly affected by both planting density and variety, but not by the interaction between density and genotype (Table 2). As the density increased, the yield tended to first increase and then decrease and peaked in the D2 group. LY66, MC670, and JK968 showed yield increases of 1.81% to 14.28%, 3.73% to 17.39%, and −3.83% to 8.37%, respectively, from D1 to D4 among the 2-yr experiments. The lower maximum yield of JK968 at high density may have resulted from severe lodging, which occurred in this variety at the vegetative stage. The optimal densities of LY66, MC670, and JK968 were calculated as 10.50 × 10^4^, 10.64 × 10^4^, and 9.93 × 10^4^ plants/ha, respectively; these densities corresponded to maximum yields of 19.48, 20.75, and 17.7 t/ha, respectively (Supplementary Fig. S3).

**Table 1.** Grain yield components and associated parameters in 3 maize varieties at several planting densities in 2019 and 2020

Year	Cultivar	Planting density	Grain yield (t/ha)	Grain weight per plant (g)	Ear # (×10^3^/ha)	Kernel #	1,000-kernel weight (g)	Pop. biomass (t/ha)	HI	Barren stalk rate (%)	Lodging rate (%)
2019	LY66	D1	18.6 ± 0.17^c^	267.8 ± 11.1^a^	73.15 ± 1.60^c^	641.89 ± 9.19^b^	417.12 ± 14.02^a^	34.38 ± 1.01^c^	0.54 ± 0.01^a^	0 ± 0^b^	…
		D2	19.8 ± 0.7^bc^	206.5 ± 7.9^bc^	100.00 ± 2.78^b^	563.85 ± 23.95^de^	381.55 ± 8.00^b^	40.30 ± 1.34^b^	0.49 ± 0.00^b^	3.56 ± 1.48^a^	…
		D3	19.5 ± 1.0^bc^	197.8 ± 4.7^c^	112.04 ± 1.60^a^	535.96 ± 5.93^e^	369.01 ± 7.22^bc^	39.07 ± 0.98^b^	0.50 ± 0.02^b^	4.72 ± 0.06^a^	…
	MC670	D1	19.8 ± 0.9^bc^	270.9 ± 13.4^a^	76.85 ± 3.21^c^	675.10 ± 19.17^a^	401.13 ± 9.98^a^	35.43 ± 0.26^c^	0.56 ± 0.02^a^	−1.19 ± 2.06^b^	…
		D2	21.4 ± 0.6^a^	227.0 ± 16.6^b^	103.70 ± 1.60^b^	605.20 ± 15.04^c^	371.12 ± 5.37^bc^	42.79 ± 0.13^a^	0.50 ± 0.01^b^	3.44 ± 1.46^a^	…
		D3	20.8 ± 1.2^ab^	206.9 ± 9.6^bc^	114.81 ± 6.42^a^	576.43 ± 21.94^d^	358.94 ± 3.06^c^	40.96 ± 1.34^ab^	0.51 ± 0.02^b^	3.88 ± 2.68^a^	…
2020	LY66	D1	17.5 ± 0.3^cde^	228.4 ± 9.0^a^	75.93 ± 1.60^g^	569.20 ± 14.06^bc^	401.19 ± 8.92^a^	32.41 ± 0.28^i^	0.54 ± 0.01^a^	0 ± 0^a^	…
		D2	19.9 ± 1.1^ab^	209.3 ± 9.1^cd^	105.56 ± 2.78^e^	552.53 ± 18.23^c^	378.63 ± 4.04^bc^	38.50 ± 1.04^bcd^	0.52 ± 0.02^abc^	0 ± 0^a^	…
		D3	18.5 ± 1.6^bc^	187.3 ± 1.8^ef^	114.81 ± 1.60^cd^	524.80 ± 3.27^d^	361.13 ± 4.58^def^	37.08 ± 1.25^def^	0.50 ± 0.03^bcd^	3.10 ± 2.69^bc^	…
		D4	17.8 ± 0.6^cde^	173.6 ± 4.2^g^	124.12 ± 1.64^b^	494.13 ± 3.84^e^	351.37 ± 6.47^fg^	36.23 ± 0.69^ef^	0.49 ± 0.01^cd^	3.56 ± 1.24^bc^	…
	MC670	D1	17.9 ± 0.3^cde^	223.1 ± 10.3^ab^	73.15 ± 1.60^gh^	580.37 ± 13.35^b^	384.48 ± 14.07^b^	33.00 ± 1.18^hi^	0.54 ± 0.02^a^	0 ± 0^a^	…
		D2	21.0 ± 0.1^a^	210.4 ± 5.3^cd^	102.78 ± 0.00^ef^	571.77 ± 7.31^bc^	368.05 ± 7.21^cde^	39.64 ± 0.52^ab^	0.53 ± 0.01^ab^	0.88 ± 1.52^ab^	…
		D3	19.9 ± 1.0^ab^	197.2 ± 7.3^de^	117.59 ± 1.60^c^	559.40 ± 15.32^bc^	354.11 ± 3.01^efg^	40.16 ± 0.22^a^	0.49 ± 0.03^bcd^	0.78 ± 1.34^ab^	…
		D4	18.6 ± 0.9^bc^	175.4 ± 5.1^g^	128.70 ± 1.60^a^	512.11 ± 5.60^de^	342.43 ± 7.94^g^	39.02 ± 1.04^abc^	0.48 ± 0.01^d^	2.10 ± 2.08^abc^	…
	JK968	D1	16.8 ± 1.0^cde^	232.8 ± 9.4^a^	72.22 ± 2.78^h^	609.70 ± 8.57^a^	381.79 ± 10.18^bc^	33.85 ± 1.26^hi^	0.49 ± 0.02^bcd^	0 ± 0^a^	72.22 ± 5.09^b^
		D2	18.2 ± 0.9^bcd^	213.8 ± 13.9^bc^	101.85 ± 1.60^f^	574.77 ± 27.23^bc^	371.85 ± 8.25^bcd^	37.56 ± 0.62^cde^	0.48 ± 0.02^cd^	0.88 ± 1.52^ab^	89.17 ± 8.04^a^
		D3	16.4 ± 1.1^de^	179.0 ± 6.1^fg^	113.89 ± 2.78^d^	501.33 ± 20.16^e^	357.14 ± 10.11^efg^	35.66 ± 0.48^fg^	0.46 ± 0.03^d^	3.91 ± 1.38^c^	82.05 ± 2.22^ab^
		D4	16.1 ± 1.6^e^	161.1 ± 7.8^h^	125.93 ± 1.60^ab^	466.67 ± 22.43^f^	345.18 ± 0.74^g^	34.29 ± 0.70^gh^	0.47 ± 0.04^d^	4.21 ± 2.06^c^	87.50 ± 6.61^a^

The data are presented as the mean ± Sd from 3 biological replicates parameter for each variety and year. The lowercase letters indicate statistical significance groups at *P* < 0.05 (2-way ANOVA). Pop. biomass, population-scale biomass accumulation at maturity; HI, harvest index.

**Table 2.** Effects of maize variety and planting density on yield and related parameters in 2019 and 2020

Variable	Effect of variety (*V*) in 2019	Effect of planting density (*D*) in 2019	Effect of *V* × *D* in 2019	Effect of *V* in 2020	Effect of *D* in 2020	Effect of *V* × *D* in 2020
Grain yield	14.3^a^	5.1^b^	0.09	17.9^a^	9.8^a^	0.68
Grain weight per plant	1.5	44.8^a^	0.12	1.6	117.7^a^	3.1^b^
Ear number	5.171^b^	232.436^a^	0.043	3.989^b^	1277.445^a^	3.234^b^
Kernel number	26.475^a^	66.983^a^	0.12	9.322^a^	99.089^a^	9.006^a^
1,000-kernel weight	7.686^b^	37.703^a^	0.191	6.304^a^	48.637^a^	0.585
Pop. biomass	14.341^a^	70.906^a^	0.751	29.13^a^	68.53^a^	6.953^a^
HI	2.34	19.3^a^	0.13	11.23^a^	10.44^a^	0.71
Barren stalk rate	0.775	13.852^a^	0.151	2.245	9.65^a^	1.016

Effect sizes shown are the *F*-values from 2-way ANOVA. Pop. biomass, population biomass at maturity; HI, harvest index. ^a^*P* < 0.01, ^b^*P* < 0.05 (2-way ANOVA).

An analysis of the yield components indicated that increases in yield under high-density conditions were primarily due to increases in ear number per unit area. This increase counteracted the significant decreases in kernel number per plant and 1,000-kernel weight. Accordingly, high planting density resulted in significant per-plant yield decreases, especially for JK968. The barren stalk rate also increased along with the planting density, with the highest rate in JK968, followed by LY66, and then MC670. Population biomass accumulation at maturity showed a similar tendency, with average increases (mean values of D2/D3/D4 to D1, among the 2-yr experiments) of 15.18%, 19.29%, and 5.89% in LY66, MC670, and JK968, respectively. Furthermore, increased planting density mainly caused significant biomass increases in Layer II, with average increases (mean values of D2/D3/D4 to D1, among the 2-yr experiments) of 15.51%, 22.30%, and 4.42% in LY66, MC670, and JK968, respectively (Supplementary Fig. S4). However, the harvest index decreased with planting density.

### Effects of planting density on per-plant biomass accumulation and transfer in each canopy layer

**Figure 1.**
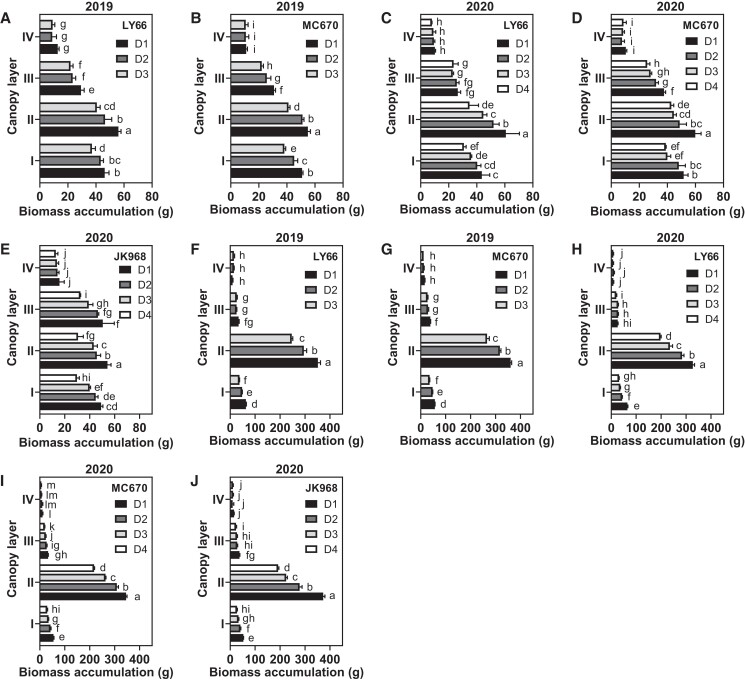
Biomass accumulation in each maize canopy layer among plants grown at several planting densities. **A to E)** Biomass accumulation at the silking stage for **A**) LY66 in 2019, **B**) MC670 in 2019, **C**) LY66 in 2020, **D**) MC670 in 2020, and **E**) JK968 in 2020. **F to J**) Biomass accumulation at the maturity stage for **F**) LY66 in 2019, **G**) MC670 in 2019, **H**) LY66 in 2020, **I**) MC670 in 2020, and **J**) JK968 in 2020. D1 to D4 represent 75,000, 105,000, 120,000, and 135,000 plants/ha, respectively. The lowercase letters indicate statistical significance groups at *P* < 0.05 (2-way ANOVA with post hoc Lsd test). The data are presented as the mean ± Se from 3 or 4 biological replicates per group.

**Figure 2.**
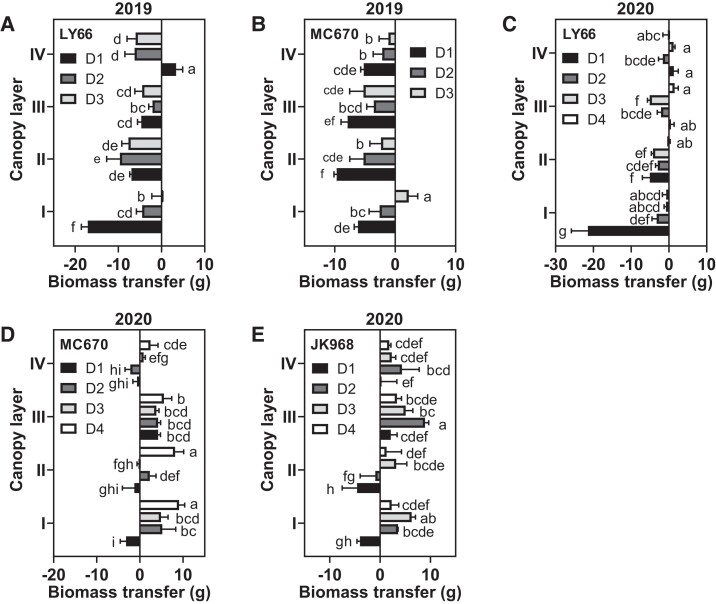
Biomass transfer in each maize canopy layer among plants grown at several planting densities. **A to E)** Biomass transfer before the silking stage in (**A**) LY66 in 2019, (**B**) MC670 in 2019, (**C**) LY66 in 2020, (**D**) MC670 in 2020, and (**E**) JK968 in 2020. A negative value indicates that the DW was higher at maturity than at the silking stage. The transfer amount was calculated from biomass accumulation per plant. D1 to D4 represent 75,000, 105,000, 120,000, and 135,000 plants/ha, respectively. The lowercase letters indicate statistical significance groups at *P* < 0.05 (2-way ANOVA with post hoc Lsd test). The data are presented as the mean ± Se from 3 or 4 biological replicates per group.

We next compared per-plant biomass accumulation between specific layers of the canopy. Biomass accumulation was most abundant in Layer II, followed by Layer I. Increases in planting density caused pronounced decreases in per-plant biomass accumulation; across cultivars and planting years, the average decreases (mean values of D2/D3/D4 to D1) were 18.20% and 24.31% in Layers I and II, respectively, at the silking stage and 35.17% and 26.92%, respectively, at the maturity stage (Fig. 1). Reductions in biomass accumulation in Layers I and II were greatest in JK968, followed by LY66 and then MC670. Increasing the planting density also increased the total biomass transfer, with greater positive effects observed in Layers I and II (averaging 11.36 and 3.95 g, respectively) than in Layers III and IV (1.44 and −0.04 g, respectively; Fig. 2). Overall, biomass transfer was greatest in MC670 and lowest in LY66. These findings suggested that biomass accumulation and transport in the lower canopy were of great importance to yield formation under high-density planting conditions.

### Effects of planting density on photosynthetic characteristics in each canopy layer

**Figure 3.**
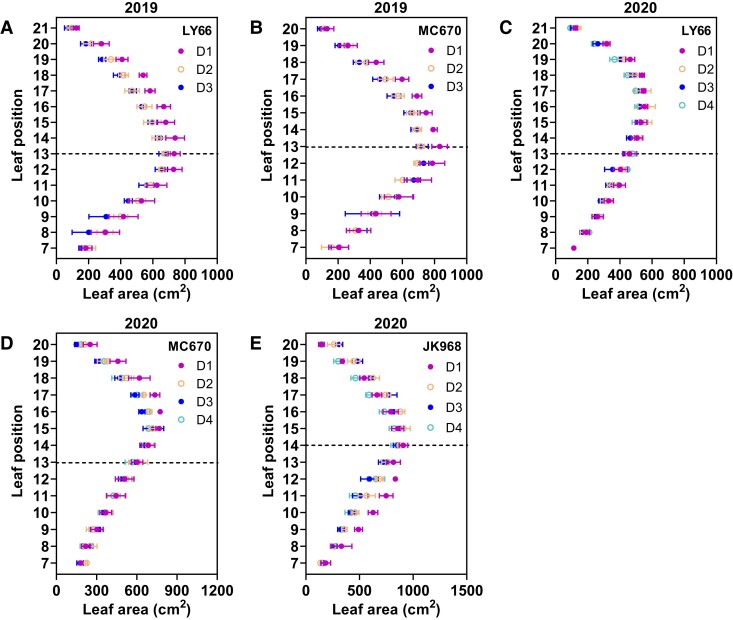
Leaf area at each leaf position among plants grown at several planting densities. **A to E)** Green leaf area at each leaf position at the silking stage in (**A**) LY66 in 2019, (**B**) MC670 in 2019, (**C**) LY66 in 2020, (**D**) MC670 in 2020, and (**E**) JK968 in 2020. The first visible complete leaf was the seventh leaf from the bottom at the silking stage. The numbers 7 to 21 indicate the 7th to 21st leaves, respectively, from the bottom of the plant. The black dotted lines represent the ear position. D1 to D4 correspond to 75,000, 105,000, 120,000, and 135,000 plants/ha, respectively. The data are presented as the mean ± Se from 3 biological replicates per group.

For individual leaves, the total area is an important indicator of photosynthetic capacity. We therefore compared the single-leaf area at each leaf position in maize plants grown at each planting density. The leaf area increased gradually with leaf position in the lower portion of the plant, peaked in the middle of the plant, and then gradually decreased with leaf position in the upper plant (Fig. 3). LY66 and MC670 plants showed similar patterns of vertical leaf area distribution across planting densities. For example, LY66 and MC670 showed significant reductions in leaf area in Layers III and IV as the planting density increased; however, in JK968, reductions in leaf area occurred in Layers I and II (Fig. 3, Supplementary Fig. S5). A further analysis of the average leaf length and average leaf width in each layer was done; in Layers I and II, LY66 and MC670 showed an increment in leaf length and a slight reduction in leaf width; in Layers III and IV, LY66 and MC670 showed a reduction in leaf length and a great reduction in leaf width. However, JK968 showed the opposite trend with these 2 varieties (Supplementary Table S1). Furthermore, the spatial density of leaf area (SDLA) generally increased with the layer number in all 3 varieties under high planting density. The greatest increases in Layer III were found in JK968 plants (Supplementary Fig. S6). These increases were associated with decreased fractional interception of photosynthetically active radiation (FIPAR) in Layer II among JK968 plants (Supplementary Fig. S7A) and indicated poor light transmission from the top to the bottom of the plant canopy (Supplementary Fig. S7B). In addition, the red to far-red ratio (R/FR) was significantly decreased in Layers I to III of LY66 and JK968 plants, but not in Layers I and II of MC670 plants (Supplementary Fig. S7C). Thus, the light quality was superior in the lower canopy layers of MC670 compared with those of LY66 or JK968.

**Figure 4.**
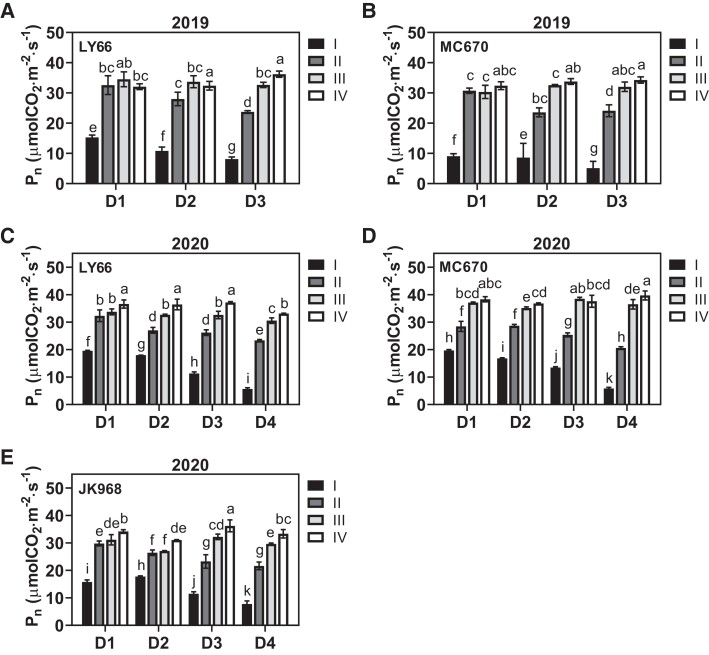
Net photosynthesis (*P*_n_) in the leaves of each canopy layer among plants grown at several planting densities. **A to E)***P*_n_ at the silking stage in (**A**) LY66 in 2019, (**B**) MC670 in 2019, (**C**) LY66 in 2020, (**D**) MC670 in 2020, and (**E**) JK968 in 2020. D1 to D4 represent 75,000, 105,000, 120,000, and 135,000 plants/ha, respectively. The lowercase letters indicate statistical significance groups at *P* < 0.05 (2-way ANOVA with post hoc Lsd test). The data are presented as the mean ± Se from 3 or 4 biological replicates per group.

**Figure 5.**
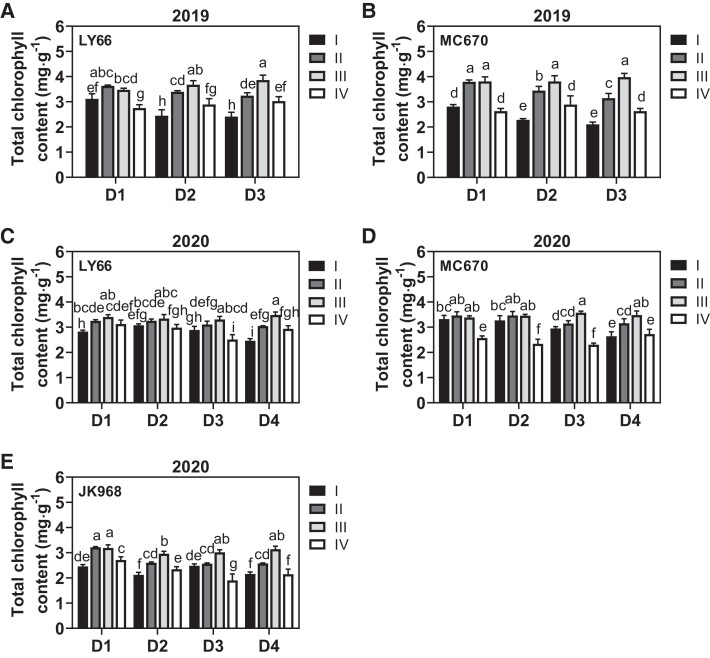
Total chlorophyll contents in leaves from each canopy layer among plants grown at several planting densities. **A to E)** Total chlorophyll contents in leaves at the silking stage in (**A**) LY66 in 2019, (**B**) MC670 in 2019, (**C**) LY66 in 2020, (**D**) MC670 in 2020, and (**E**) JK968 in 2020. D1 to D4 represent 75,000, 105,000, 120,000, and 135,000 plants/ha, respectively. The lowercase letters indicate statistical significance groups at *P* < 0.05 (2-way ANOVA with post hoc Lsd test). The data are presented as the mean ± Se from 3 biological replicates per group.

As a consequence of low light interception and its negative effects on leaf area, increasing the planting density significantly reduced the net photosynthetic rate (*P*_n_) of leaves in Layers I and II but not in Layers III or IV (Fig. 4). Moreover, high planting density led to a greater *P*_n_ reduction among leaves in Layer I (mean = 32.47%) than in Layer II (mean = 19.71%). A similar trend was observed for the total chlorophyll content (Fig. 5), which increased more in Layer II than in Layer I, peaked in Layer III, and then decreased again in Layer IV. The decreased *P*_n_ and total chlorophyll contents of MC670 were reduced by a smaller margin in the lower canopy layers than in the other varieties.

### Effects of planting density on maize ear development

The maize ear length, diameter, and bald tip length were measured for each variety and treatment group at maturity. The ear length and diameter decreased along with planting density, whereas the bald tip length increased. JK968 was the most sensitive to increased planting density with respect to the increase in bald tip length (Supplementary Fig. S8). Furthermore, assessments of tassel and ear developmental processes indicated that increased planting density resulted in plant growth delays. Specifically, the silking stage was delayed by 2 to 3, 3 to 6, and 3 to 8 d in the D2 to D4 treatments, respectively, compared with D1. However, planting density had a smaller effect at the tassel stage than at the silking stage, leading to a longer ASI among plants grown under high-density conditions (Supplementary Table S2).

**Figure 6.**
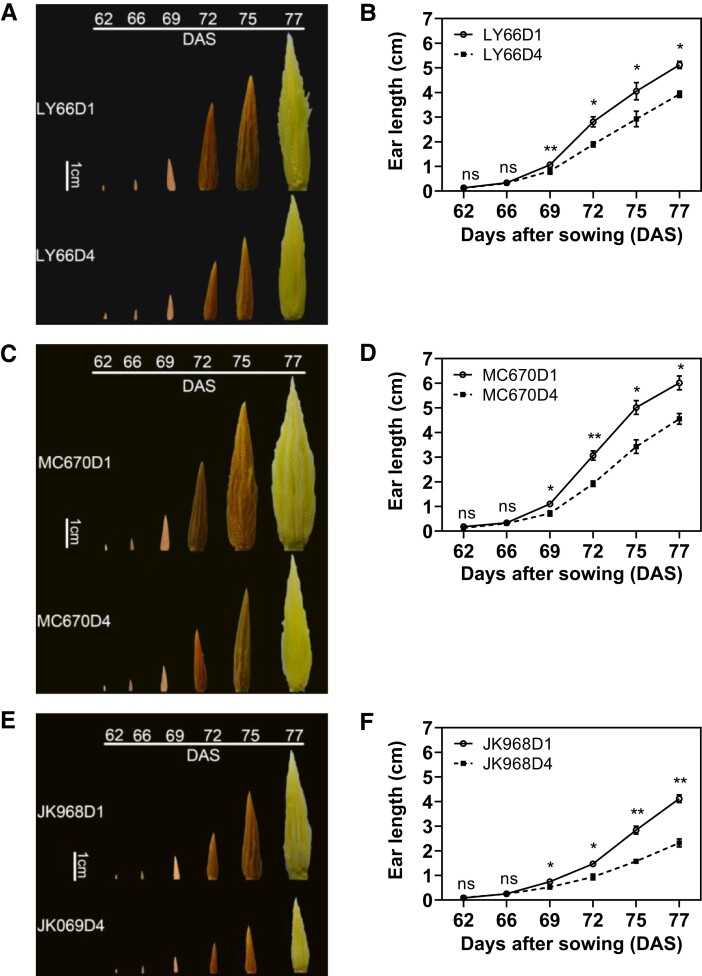
Young ear development among plants grown at several planting densities. **A, C, E)** Representative (**A**) LY66, (**C**) MC670, and (**E**) JK968 ears at several time points after sowing in 2020. Images were digitally extracted for comparison. **B, D, F)** Quantification of ear length over time for (**B**) LY66, (**D**) MC670, and (**F**) JK968 plants. Scale bar = 1 cm. D1, 75,000 plants/ha; D4, 135,000 plants/ha. *P < 0.05, **P < 0.01 (Student's *t*-test). ns, not significant. The data are presented as the mean ± Se from 4 biological replicates per group.

**Figure 7.**
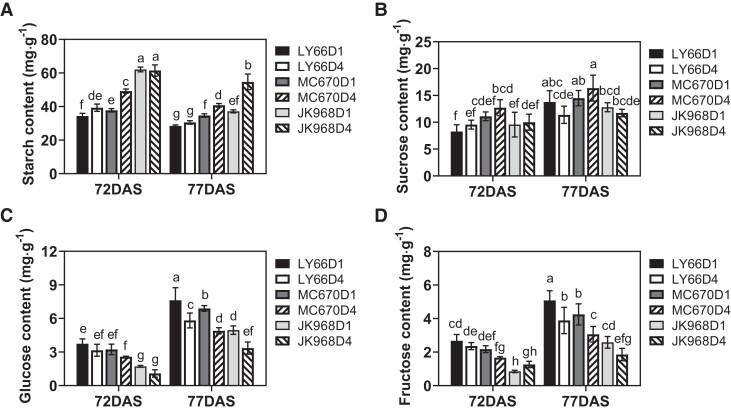
Levels of starch, sucrose, glucose, and fructose in young maize ears from plants grown at several planting densities. **A to D)** Levels of (**A**) starch, (**B**) sucrose, (**C**) glucose, and (**D**) fructose. Samples were analyzed at 72 and 77 DAS in 2020. D1, 75,000 plants/ha; D4, 135,000 plants/ha. The lowercase letters indicate statistical significance groups at *P* < 0.05 (2-way ANOVA with post hoc Lsd test). The data are presented as the mean ± Se from 3 or 4 biological replicates per group, each of which consisted of pooled samples from at least 3 plants.

Increased planting density did not appear to affect tassel development or initial ear differentiation (Fig. 6, Supplementary Fig. S9), although ear development (as measured by ear length) lagged significantly in D4 compared with D1 (Fig. 6). Stagnation in ear development under dense planting conditions was more severe as the ears grew; ear lengths in the D4 treatment were decreased by 23.77% to 35.09% compared with D1 at 69 d after sowing (DAS), but by 23.18% to 43.67% at 77 DAS (Fig. 6). Furthermore, starch content decreased over time, whereas sucrose, glucose, and fructose contents increased as the ears grew. Starch content was significantly higher under D4 than under D1 conditions, especially at 77 DAS. Levels of glucose and fructose in the ear were significantly decreased (by 15.57% to 36.86% and 11.79% to 48.95%, respectively) in D4 compared with D1 plants (Fig. 7). Overall, ear length, glucose levels, and fructose levels were most strongly impacted by planting density in JK968 plants, followed by MC670, then LY66.

### Relationships between grain yield and photosynthetic parameters at each planting density

**Figure 8.**
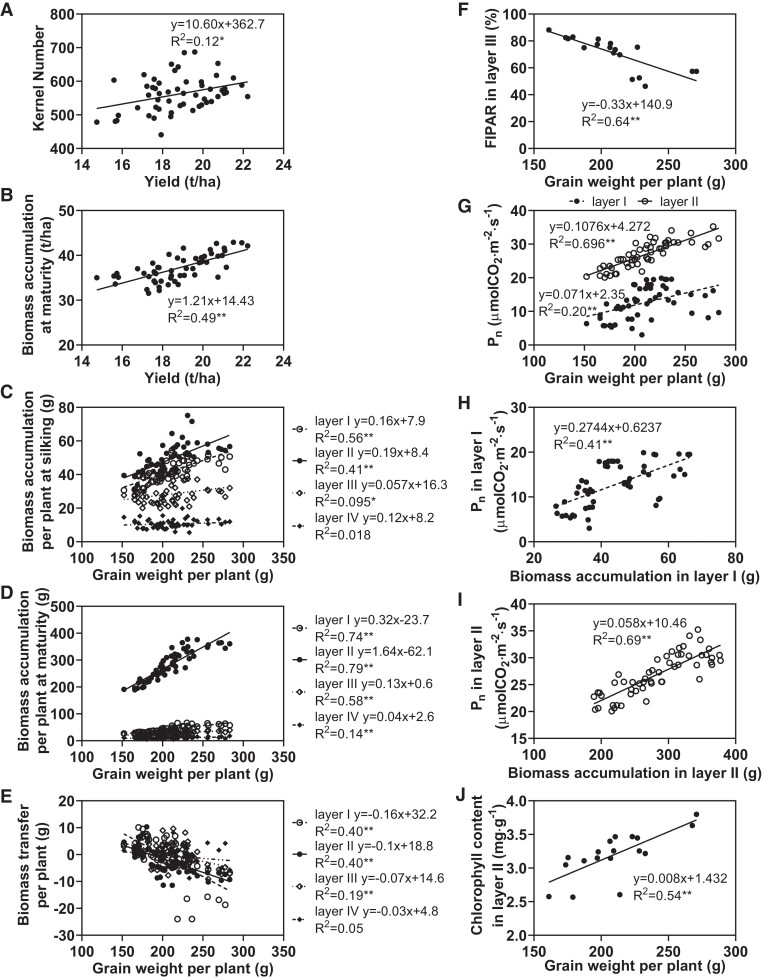
Correlation of yield components with physiological parameters at each canopy layer among plants grown at several planting densities. **A and B)** Correlation of yield with (**A**) kernel number and (**B**) biomass accumulation at maturity. **C to G)** Correlation of grain weight per plant with (**C**) biomass accumulation per plant at silking, (**D**) biomass accumulation per plant at maturity, (**E**) biomass transfer per plant, (**F**) FIPAR in Layer III, and (**G**) *P*_n_. **H and I)** Correlation of biomass accumulation per plant at maturity in (**H**) Layer I and (**I**) Layer II with *P*_n_. **J)** Correlation of grain weight per plant with chlorophyll content in Layer II. *P < 0.05, **P < 0.01 (Pearson correlation analysis). **A to E)** and **G to I**) A total of 54 replicates, each point represents 1 replicate of 1 planting density and 1 variety and 1 yr. **F** and **J**) A total of 18 replicates, each point represents 1 planting density of 1 variety and 1 yr. **A and B)** The data are based on the entire canopy. **C to J)** The data are based on different canopy layers.

Correlation analyses were conducted to comprehensively investigate the relationships between grain yield and physiological parameters associated with biomass accumulation and photosynthesis at each planting density (Fig. 8, Supplementary Figs. S4 and S8, Supplementary Data Set 1). Grain yield was positively correlated with kernel number (Fig. 8A). Moreover, kernel number was significantly correlated with ear length, ear diameter, and bald tip length (Supplementary Fig. S8, G to I). Population-scale biomass accumulation was positively correlated with grain yield (Fig. 8B), and population-scale biomass accumulation in Layers I and II was positively correlated with grain yield (Supplementary Fig. S4F). Furthermore, per-plant biomass accumulation in Layers I and II was positively correlated with grain weight per plant (Fig. 8, C and D), and biomass transfer in Layers I and II was negatively correlated with grain weight per plant (Fig. 8E). FIPAR in Layer III was negatively correlated with grain weight per plant (Fig. 8F), and *P*_n_ in Layers I and II was positively correlated with grain weight per plant (Fig. 8G) and biomass accumulation per plant at maturity in Layers I and II (Fig. 8, H and I). Total chlorophyll content in Layer II was positively correlated with grain weight per plant (Fig. 8J). Overall, grain yield and per-plant grain yield were dependent on photosynthetic parameters in Layers I and II.

## Discussion

Substantial increases in maize yield have been achieved in recent decades due to advances in agricultural technologies and breeding approaches. Increasing the planting density is one of the most important crop management strategies identified for increasing maize yield (Sher et al. 2017; Zhang et al. 2020). This was clearly demonstrated in 87 farm experiments undertaken in China from 2017 to 2020, which showed yield gains of 7.3% in response to increased planting density (Luo et al. 2023). However, yield increases associated with high planting density are not infinite; each variety performs best at an optimal density, beyond which yield declines (Deng et al. 2012; Mastrodomenico et al. 2018; Wei et al. 2020). In the present study, total yield was generally increased by dense planting conditions, peaking at D2 for each variety (Table 1). Yield increases in response to high planting density were greatest in MC670, followed by LY66, then JK968. These high yields resulted from the combined effects of increases in the total ear number, kernel number, and 1,000-kernel weight.

Photoassimilation is the foundational basis of plant productivity and biomass production (Gaju et al. 2016), with leaves serving as the primary organs responsible for light interception and photosynthesis (Chen et al. 2019). We found that increases in the planting density decreased the light interception area, primarily in Layer II or III (Supplementary Fig. S5), and increased the SDLA (Supplementary Fig. S6). These changes reduced the photosynthetic rate and, thus, biomass production (Fig. 1). However, population biomass accumulation at maturity showed increases of varying degrees along with density (Table 1, Supplementary Fig. S4). Biomass accumulation varied between varieties but was generally highest in MC670 and lowest in JK968. Notably, biomass accumulation at both the population and single-plant scales, especially in the lower canopy was positively correlated with grain yield and grain weight per plant (Fig. 8, Supplementary Fig. S4F). A recent study demonstrated that the proportion of dry matter accumulation after silking increases, whereas the dry matter transfer rate decreases, in high-yield maize (Liu et al. 2023). In this study, we found that biomass accumulation after silking was decreased, but that biomass transfer was increased; this was especially true in MC670 in 2020, which showed relatively higher biomass transfer at the bottom layer (Fig. 2). We, therefore, propose that the strong biomass accumulation and biomass redistribution capacity of maize plants at high density, particularly below the ear, can maintain high plant productivity.

Under dense planting conditions, the spatial distribution of the leaf area is known to affect light interception and utilization (Perez et al. 2019); the altered light environment of the lower canopy (i.e. reduced light intensity and/or altered spectral composition), rather than normal aging, causes decreased efficiency among shaded leaves (Collison et al. 2020). We observed that increased planting density generally increased the SDLA in all 4 canopy layers (Supplementary Fig. S6), implying that there was weaker canopy transmission and poor light quality under high-density conditions (Supplementary Fig. S7, B and C). Moreover, increased planting density reduced the per-leaf area in Layers II and III (LY66 and MC670) or Layers I and II (JK968; Fig. 3, Supplementary Fig. S5). This distribution of leaf area ultimately led to great increases in SDLA within Layer III of JK968 plants, contributing to higher and lower FIPAR values in Layers III and II, respectively (Supplementary Fig. S7A). Furthermore, the observed leaf area patterns allowed more photosynthetically active radiation (PAR) to reach the lower layers in LY66 and MC670, resulting in higher photosynthetic rates and increased chlorophyll contents in Layers I and II of these plants than in JK968 (Figs. 4 and 5); those photosynthetic parameters were significantly positively correlated with per-plant yield (Fig. 8).

Numerous prior publications have focused on changes in physiological function along the vertical gradient within a canopy (Ciampitti and Vyn 2013; Chen et al. 2015; Song et al. 2018; Odorico et al. 2019; Xu et al. 2021). Such studies have generally shown that traits related to photosynthetic capacity remain high in middle-canopy leaves, such as the ear leaf and adjacent leaves (Escobar-Gutiérrez and Combe 2012; Song et al. 2018; Xu et al. 2021). We here found that chlorophyll content was highest in Layers III and II, which were near the ear position, consistent with previous reports (e.g. Li et al. 2019). Our results indicated that *P*_n_ decreased consistently from the top to the bottom layers (Fig. 4), which may have been related to leaf senescence and/or low light conditions in the lower canopy (Escobar-Gutiérrez and Combe 2012; Hikosaka et al. 2016). High planting density places a great number of leaves in a shaded environment, which can restrict leaf development and photosynthesis, ultimately limiting biomass and yield (Raza et al. 2019). Increased planting density primarily reduced *P*_n_ and pigment contents among leaves in Layers I and II (Figs. 4 and 5). This implied that the functional traits of leaves in the lower layers were more severely compromised than those in the upper layers. Thus, increasing the planting density reduced biomass accumulation by a greater margin in Layers I and II than in Layers III or IV. Statistical analysis revealed positive associations of ear weight and per-plant biomass accumulation with biomass accumulation, *P*_n_, and total chlorophyll contents in Layers I and II specifically (Fig. 8), similar to earlier findings (Zhao et al. 2015). Collectively, these results suggested that photosynthetic production in Layers I and II was the primary contributor to increases in grain yield due to high planting density.

Both planting density and maize variety had significant impacts on yield, kernel number, and 1,000-kernel weight (Table 2). Under high-density planting, kernel number generally contributes more to yield variations than any related parameters do, including 1,000-kernel weight (Andrade et al. 1999). A previous study reported that kernel number is mainly determined by floret number, which varies by genotype (Cárcova et al. 2000). However, specific conditions (e.g. planting density) can also affect kernel number by affecting floret degradation and kernel set (Rossini et al. 2011). In the present study, increasing planting density delayed the time to silking by up to 2 to 8 d; this corresponded to increased ASI and decreased ear length (Fig. 6, Supplementary Fig. S8 and Table S2).

Many studies have shown that kernel number is also affected by photosynthetic capacity and photoassimilate accumulation (Otegui and Bonhomme 1998; Cui et al. 2015). Carbohydrates, including sugars and starches, are dependent on photoassimilates from source organs and are the main sources for reproductive development (Macneill et al. 2017; Gustin et al. 2018). Sugars also act as signaling molecules, regulating the expression of various genes involved in metabolic pathways and cellular functions (Valluru 2015; Hans-Wilhelm et al. 2018; Ruben et al. 2018). Low photosynthetic capacity in the leaves and the resulting insufficient assimilate supply can cause poor ear development, exacerbating yield losses (Pawar and Rana 2019; Hu et al. 2022). In the present study, increasing the planting density increased starch content in the ears, but reduced levels of soluble sugars, especially glucose and fructose (Fig. 7). This suggested that increased planting density may have reduced ear metabolism, inhibiting development and thus yield. Moreover, due to the role of glucose as the main component of cell wall polysaccharides, low levels of this sugar may reduce the cellular growth rate (Shao et al. 2018). The observed low glucose levels may, therefore, have been responsible for reductions in ear length and diameter (Fig. 6 and Supplementary Fig. S8), which were significantly positively correlated with kernel number (Supplementary Fig. S8, G to I). In addition, we found that increasing the planting density significantly impacted the ear, but not the tassel (Fig. 6, Supplementary Fig. S9 and Table S2). We hypothesized that this discrepancy was due to more intense intraspecies competition during stages in which the ear was developing [V9 to VT (tasseling stage)] than during stages in which the tassel was developing (V6 to V12). Notably, the tassel is also located at the top of the canopy, which shows fewer density-dependent effects.

**Figure 9.**
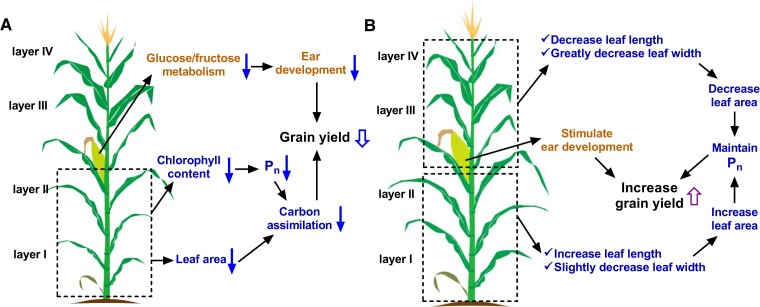
A schematic diagram showing the physiological mechanisms of yield losses or gains among plants grown at several planting densities. Representations of (**A**) reduced and (**B**) increased grain yield under high-density planting conditions. Increased planting density reduces the photosynthetic rate and leaf area in the lower canopy layer, thereby reducing assimilate accumulation. Moreover, increased planting density affects the glucose and fructose contents of young ears, impairing ear development. This ultimately reduces kernel number per ear and per-plant kernel weight, resulting in per-plant yield losses. Optimizing leaf morphology in the canopy layers in response to increased planting density could improve the photosynthetic rate and stimulate ear development, increasing yield. The dashed boxes represent the parts below and above the ear in the plant. The processes named in turmeric, blue, and black correspond to ear development, carbon metabolism, and yield, respectively. The black arrows represent the indication. The blue arrows (regular solid arrows and outline arrow) and purple outline arrow represent decreases and increases, respectively, in the indicated processes.

In conclusion, the results of this study indicated that high planting density was associated with decreased photosynthetic capacity of leaves within the lower canopy, which led to decreased biomass production. Furthermore, increased planting density suppressed ear development. These influences on both the leaves and the ears resulted in significant per-plant yield loss. Thus, strategies for maximizing grain yield under high-density planting conditions should focus on 2 key areas: optimizing the canopy structure to maintain high photosynthetic efficiency in the lower canopy leaves and stimulating ear development (Fig. 9). Moreover, we characterized the maize ideotype for high planting density, that the leaf length and width should be reduced in the upper canopy, facilitating light penetration into the lower canopy, to further benefit photosynthesis in the lower canopy with increased leaf length and slightly decreased leaf width. Our study not only provides mechanistic insights into biochemical processes affecting grain yield under high-density conditions, but also establishes critical target traits for future maize breeding efforts, ultimately contributing to the development of high-yield maize and thus food security.

## Materials and methods

### Plant materials and experimental design

Field experiments were conducted at the Wangtai Experimental Station of Ningxia Academy of Agricultural and Forestry Sciences, Ningxia, China (106°14′E, 38°14′N). Experiments were carried out during the growing seasons of 2019 and 2020 (Supplementary Fig. S1). The field site was located in the arid and semi-arid region of northwest China, in the irrigated zone for spring maize (*Z. mays* L.). Precipitation was monitored with a Watchdog portable-meteorological station (Watchdog 2900ET, Spectrum Technologies Inc., Aurora, IL, USA). The soil type was light sierozem, with 73.4 mg/kg alkali-hydrolyzable nitrogen, 66.0 mg/kg available phosphorus (Olsen-P), 313.8 mg/kg available potassium (NH_4_Ac-K), 1.46 g/kg total nitrogen, and 17.3 g/kg organic matter in the 0 to 20 cm soil layer. After harvesting in 2018, 150 kg/ha diammonium phosphate (containing 18% N and 20% P) was applied in combination with deep plowing and winter irrigation. Base fertilizers were applied prior to sowing, containing 225 kg N/ha (urea), 300 kg P_2_O_5_/ha (super phosphate), and 150 kg K_2_O/ha (potassium sulfate). Additional fertilizer [225 kg N/ha (urea)] was applied at the silking stage. Diffuse irrigation was conducted 4 times during the growing period.

The maize cultivars “Liangyu 66” (LY66), “Jingke 968” (JK968), and “MC670” were selected for the field experiments due to their high yield under different planting densities. Maize was sown at 3 densities (D1 to D3) on April 25 2019 and at 4 densities (D1 to D4) on April 15 2020. Rows were spaced 60 cm apart; spacing within each row was adjusted to reach the appropriate density. Three experimental plots per treatment with the split–split plot design were conducted each year, with planting density and cultivars as the main plot and subplot, respectively. Weeds, diseases, and pests were well-controlled by applying pesticides within the plots.

For canopy profiling, aerial plant tissues were vertically divided into 4 layers based on the ear position; the upper and lower parts of the ear were divided into 2 layers (Supplementary Fig. S2). All organs were measured in their natural state within the canopy. The 4 canopy layers were designated I to IV from the bottom of the plant to the top. The leaf located in the middle of each layer was selected as the representative leaf for gas exchange and pigment measurements, as described below.

### Grain yield and lodging rate measurements

The 1,000-kernel weight and kernel number were measured at physiological maturity. Plants in the middle of each plot were selected for these measurements, and 20 ears per plot were gathered. Measurements were taken after the ears were air dried. To measure grain yield, a 12 m^2^ region in the middle of a plot was selected; all plants within that region were harvested to form a single biological replicate. Samples were dried, and the grain moisture content was standardized (14%) before yield was calculated. Three biological replicates were analyzed per treatment group in 2019 and 2020. The plant lodging rate was calculated as the percentage of lodged plants out of the total plant number per plot. Four replicates were performed for each treatment in 2020.

### Biomass accumulation and transfer measurements

Plant aerial tissues were vertically divided into 4 layers. Leaves, stems (including the internodes, sheaths, and tassels), and ears (including the husks, cobs, and kernels) of each layer were collected from 3 or 4 representative plants per group at the silking and maturity stages in 2019 and 2020. Tissues were separated and oven-dried to a constant weight, which was recorded as the dry weight (DW). The sum of all parts for each plant was considered the accumulated biomass. There were 3 to 4 independent replicates per group. Biomass transfer was calculated as described by Wang et al. (2021): subtraction of the biomass accumulation in nutritional organs at maturity from the biomass accumulation in nutritional organs at the silking stage.

### Leaf area and light measurements

At the silking stage, 3 representative plants per group were selected for leaf area measurements in 2019 and 2020. Leaves at every position were measured to determine the maximum leaf width (*W*) and the leaf length (*L*). The leaf area (*S*) was then calculated as follows:


S=0.75×L×W

SDLA was calculated as the leaf area index divided by the plant height at each layer.

Light measurements were taken in each layer at 655 to 665 nm (red) and 725 to 735 nm (far red) with a SpectraPen LM500 hand-held spectrometer (Photon Systems Instruments, Drásov, Czechia) on a sunny, cloudless day. Three replicates were measured for each treatment in 2020. Using these measurements, R/FR was calculated as irradiance in the red band divided by irradiance in the far-red band. Canopy transmission was calculated as follows:


$$\mathrm{Transmission}\;(\text{\%})=\mathrm{PA}R_{n}/\mathrm{PA}R_{\mathrm{top}}\times 100$$

where PAR*_n_* is PAR in each layer and PAR_top_ is PAR above canopy. The PAR was measured for each layer at the silking stage on a clear day from 11:00 to 13:00 with a SunScan line quantum sensor (Delta-T Devices, Cambridge, UK). The interception of PAR corresponded to PAR*_n_* minus PAR*_n_*_–1_ and was then used to calculate FIPAR as follows:


$$\mathrm{FIPAR}=\mathrm{IPAR}/\mathrm{PA}R_{n}$$

### Gas exchange measurements

*P*_n_ was measured at the silking stage using the LI-6400XT portable photosynthesis system equipped with an LED leaf chamber (Li-Cor Inc., Lincoln, NE, USA). Measurements were taken for the representative leaf in each canopy layer. The photosynthetic photon flux density was assumed to be 2,000 *μ*mol photons m^−2^ s^−1^ on sunny days. Measurements were taken on 3 replicate plants in 2019 and 4 replicate plants in 2020 per group.

### Pigment measurements

In each canopy layer, the selected representative leaf from 3 plants per group was collected at the silking stage in 2019 and 2020, and frozen at −80 °C. After freezing, each leaf was homogenized via milling, then combined with 1 mL of acetone (100%). Samples were incubated in acetone at 4 °C until all pigments were removed from the leaf tissue. Samples were centrifuged for 10 min at 4 °C and 10,000 × *g*. After collection of the supernatant, samples were measured using an Ultrospec 8000PC dual-beam spectrophotometer (Biochrom Ltd., Cambridge, UK). These measurements were used to calculate the total carotenoid and chlorophyll contents in each sampled leaf as previously described (Lichtenthaler 1987). Total chlorophyll content was calculated as the sum of chlorophyll *a* and chlorophyll *b* content.

### Tassel and ear development and flowering rate

Four representative plants from D1 and D4 plots were harvested at 2 to 3 d intervals, beginning at the 10th leaf (V10) stage, to assess the tassel and ear developmental processes in 2020. The growth cone was stripped with a dissecting needle and then fixed with formaldehyde/alcohol/acetic acid solution. The tassels and ears were photographed with a DSC-WX300 digital camera (Sony Corporation, Tokyo, Japan) and a SteREO Discovery V8 stereoscopic microscope (ZEISS, Oberkochen, Germany). The tassel and ear lengths were also measured.

Before anthesis, plants within a 6 m^2^ area of each plot were labeled. The number of silking ears was then recorded after 16:00 every day. When the percentage of silking ears reached ≥60% for the first time, the plot was recorded as having reached the silking stage.

### Sucrose, glucose, fructose, and starch content measurements

Ear cones from D1 and D4 groups were harvested at 72 and 77 DAS in 2020 and then frozen at −80 °C. The materials were ground to a fine, homogeneous powder with liquid nitrogen. Sugars were extracted from 30 mg of milled ear cone per sample and measured with a sucrose/D-fructose/D-glucose detection kit (K-SUFRG) and a total starch detection kit (K-TSTA) (both from Megazyme, Bray, Ireland).

### Statistical analyses

Data were processed in Microsoft Excel 2016. Differences between groups were analyzed with a 2-way ANOVA. Lsd multiple comparison and correlation analyses were performed in SPSS 21.0 (SPSS Institute Inc., Chicago, IL, USA). Differences were considered statistically significant at *P* < 0.05. Figures were generated in GraphPad Prism 8 (GraphPad, San Diego, CA, USA).

## Supplementary Material

kiae204_Supplementary_Data

## Data Availability

The data underlying this article are available in the article and in its online supplementary materials.

## Supplementary data

The following materials are available in the online version of this article.

**Supplementary Figure S1**. Experimental site location and conditions.

**Supplementary Figure S2**. A schematic diagram of canopy layer differentiation for sampling and measurements.

**Supplementary Figure S3**. Relationships between planting density and grain yield.

**Supplementary Figure S4**. Population biomass accumulation in each maize canopy layer among plants grown at several planting densities.

**Supplementary Figure S5**. Green leaf area within each canopy layer among plants grown at several planting densities.

**Supplementary Figure S6**. Spatial density of leaf area (SDLA) in each canopy layer among plants grown at several planting densities.

**Supplementary Figure S7**. Light parameters across canopy layers among plants grown at several planting densities.

**Supplementary Figure S8**. Effects of planting density on maize ear traits at maturity.

**Supplementary Figure S9**. Tassel lengths of 3 maize cultivars among plants grown at several planting densities.

**Supplementary Table S1**. The average leaf length and average leaf width in each maize canopy layer among plants grown at several planting densities.

**Supplementary Table S2**. Days in the tasseling and silking stages among plants grown at several planting densities.

**Supplementary Data Set 1**. A summary of the level of significance (*P*-value) for correlation analyses.

## Dive Curated Terms

The following phenotypic, genotypic, and functional terms are of significance to the work described in this paper:

base CHEBI: CHEBI:22695

carbohydrates CHEBI: CHEBI:16646

ear AmiGo: PO:0020136

leaf AmiGo: PO:0025034
