# Supplementary material for: Photosynthetic capacity and assimilate transport of the lower canopy influence maize yield under high planting density
Source: Plant Physiol. 2024 Apr 9;195(4):2652–67. doi: 10.1093/plphys/kiae204 (PMC11288763; doi:10.1093/plphys/kiae204)
Supplement: kiae204_Supplementary_Data [file kiae204_supplementary_data.zip › PP2024RA00162R1_Supplementary_Data2.pdf]

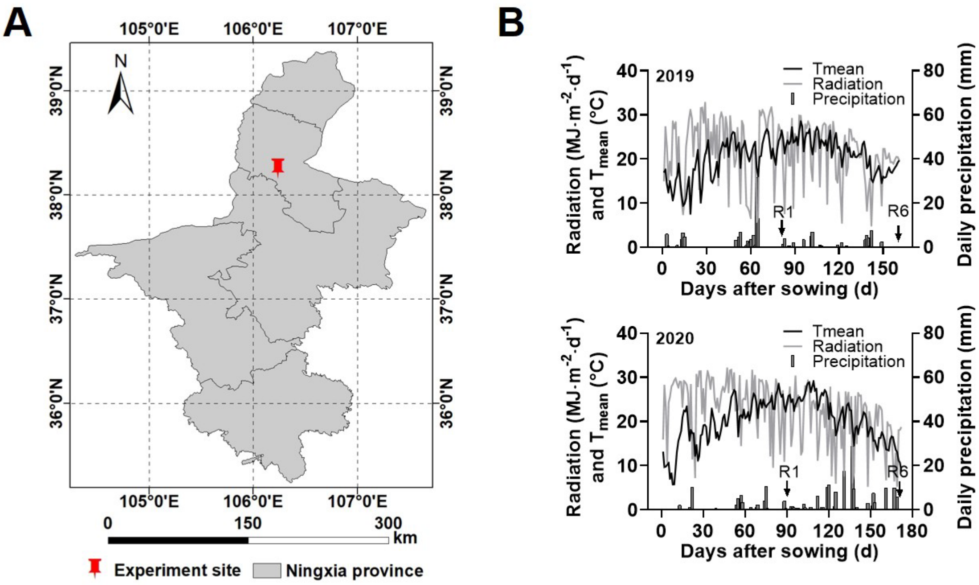

Supplemental Figure S1. Experimental site location and conditions.

(**A**) Location of the experimental site. (**B**) Meteorological data for the maize growth periods in 2019 and 2020. R1 and R6 correspond to silking and maturity stage, respectively. Radiation was measured in MJ/m^2/day; mean temperature (T_{mean}) was measured in °C; precipitation was measured in mm. Harvesting dates at R1 and R6 are indicated with arrows.

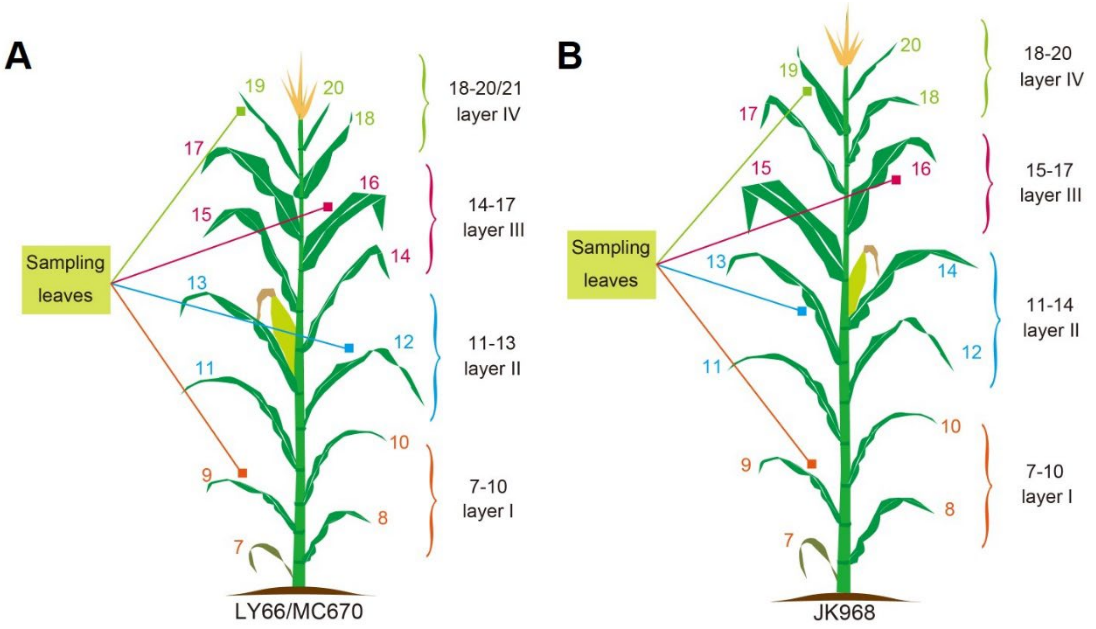

Supplemental Figure S2. Schematic diagram of canopy layer differentiation for sampling and measurements.

(**A, B**) Schematic diagram of canopy layer differentiation for (**A**) LY66, MC670 and (**B**) JK968. Numbers indicate leaf positions from the bottom to the top of the plant.

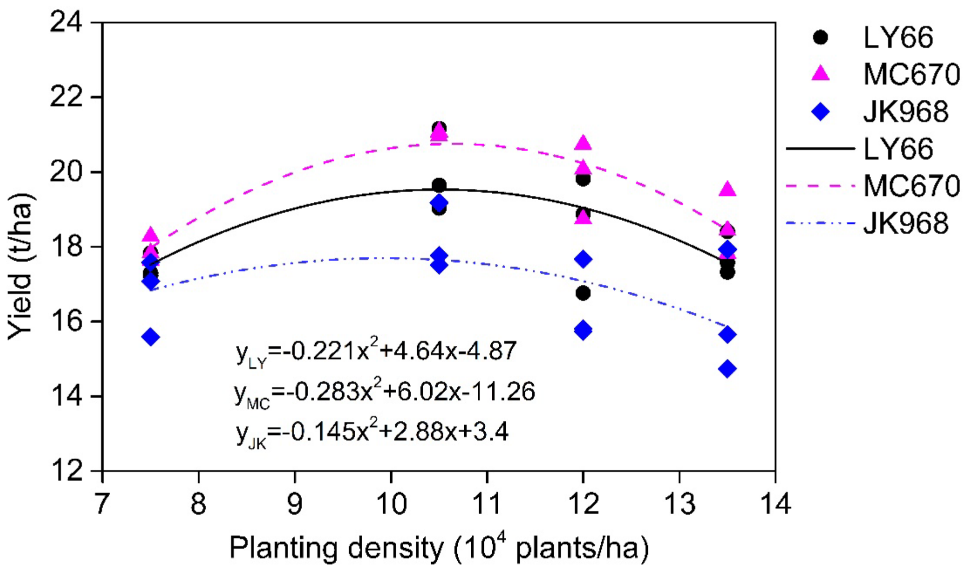

Supplemental Figure S3. Relationships between planting density and grain yield. Data were fitted to a polynomial equation for analysis and prediction of optimal planting density for maximum yield. LY, Liangyu 66; MC, MC670; JK, Jingke 968.

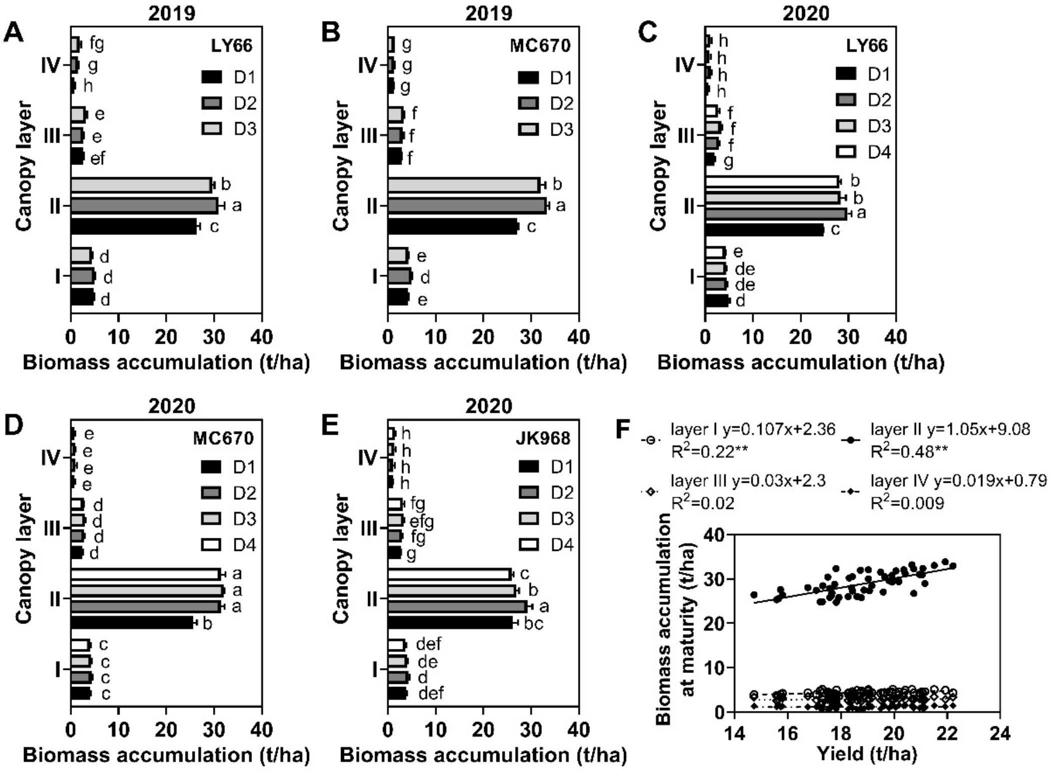

Supplemental Figure S4. Population biomass accumulation in each maize canopy layer among plants grown at several planting densities.

(**A–E**) Population biomass accumulation at the maturity stage for (**A**) LY66 in 2019, (**B**) MC670 in 2019, (**C**) LY66 in 2020, (**D**) MC670 in 2020, and (**E**) JK968 in 2020. (**F**) Correlation of population biomass accumulation in each maize canopy layer with grain yield. D1–D4 represent 75,000, 105,000, 120,000, and 135,000 plants/ha, respectively. Lowercase letters indicate statistical significance groups at $p < 0.05$ (two-way analysis of variance with post-hoc least significant difference test). Data are presented as the mean ± standard error from three or four biological replicates per group.

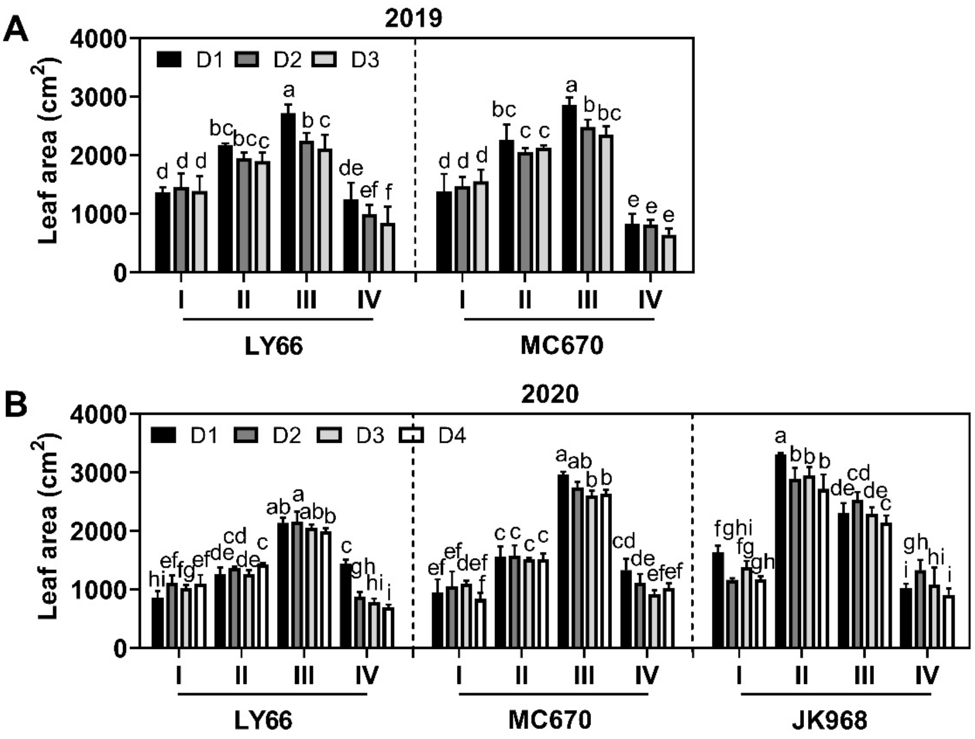

Supplemental Figure S5. Green leaf area within each canopy layer among plants grown at several planting densities.

(**A, B**) Green leaf area of leaves collected at the R1 stage in (**A**) 2019 and (**B**) 2020. Lowercase letters indicate statistical significance groups at $p < 0.05$ (two-way analysis of variance with post-hoc least significant difference test). D1–D4 correspond to 75,000, 105,000, 120,000, and 135,000 plants/ha, respectively. Data are presented as the mean ± standard error from three biological replicates per group.

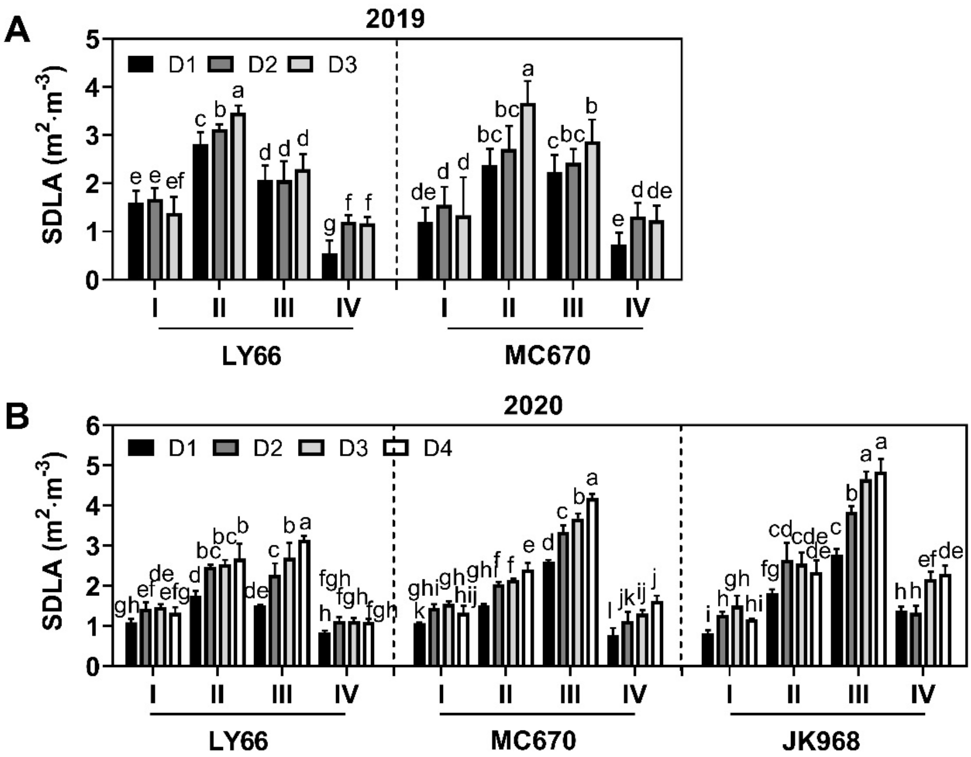

Supplemental Figure S6. Spatial density of leaf area (SDLA) in each canopy layer among plants grown at several planting densities.

(**A, B**) SDLA values for plants collected at the silking stage in (**A**) 2019 and (**B**) 2020. Lowercase letters indicate statistical significance groups at $p < 0.05$ (two-way analysis of variance with post-hoc least significant difference test). D1–D4 correspond to 75,000, 105,000, 120,000, and 135,000 plants/ha, respectively. Data are presented as the mean ± standard error from three biological replicates per group.

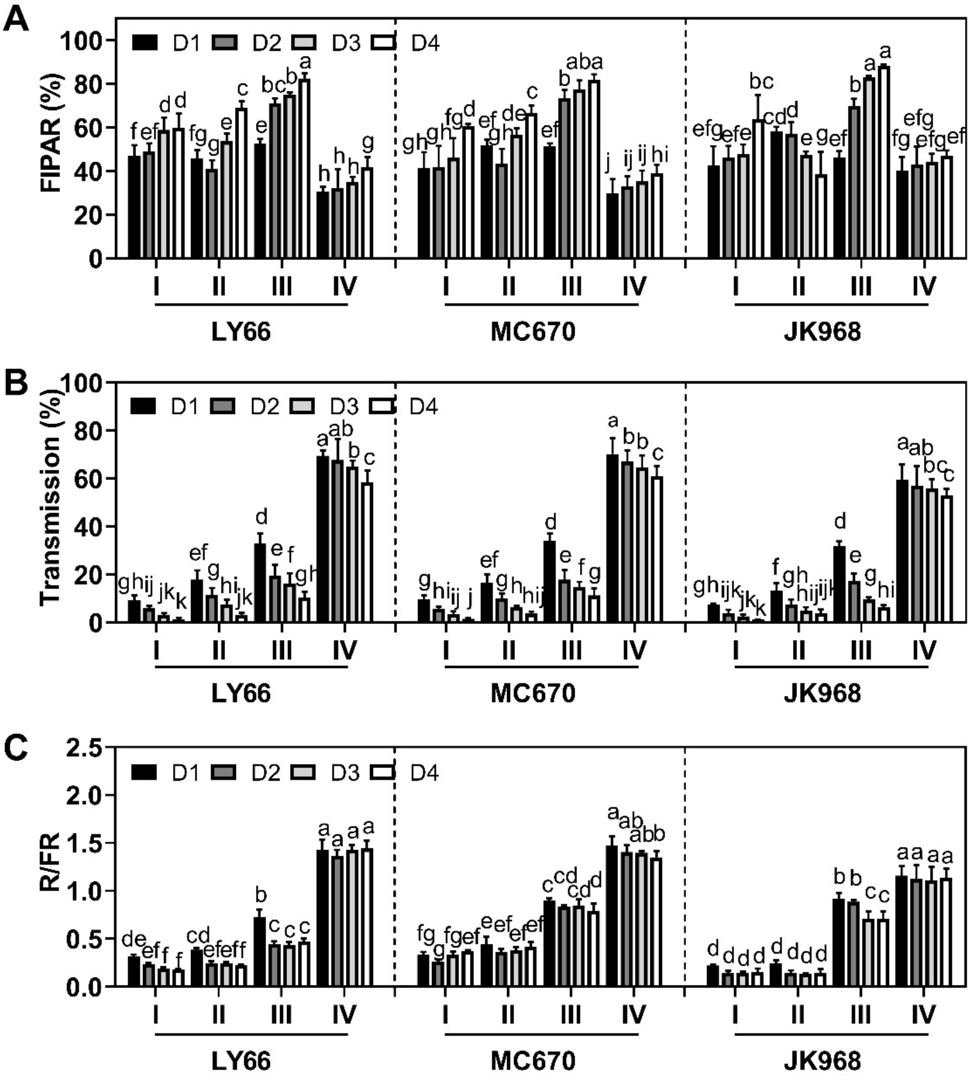

Supplemental Figure S7. Light parameters across canopy layers among plants grown at several planting densities.

(**A–C**) Measurements of the (**A**) fractional interception of photosynthetically active radiation (FIPAR), (**B**) canopy transmission, and (**C**) red to far-red ratio (R/FR) at the silking stage in 2020. Lowercase letters indicate statistical significance groups at $p < 0.05$ (two-way analysis of variance with post-hoc least significant difference test). D1–D4 correspond to 75,000, 105,000, 120,000, and 135,000 plants/ha, respectively. Data are presented as the mean ± standard error from three biological replicates per group.

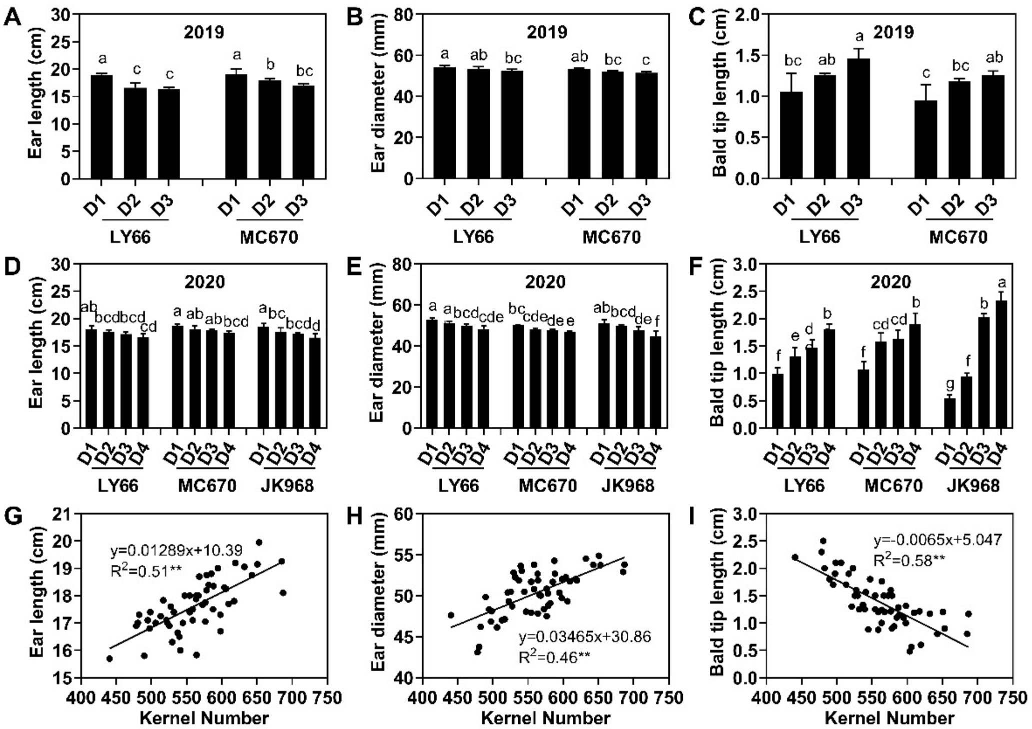

Supplemental Figure S8. Effects of planting density on maize ear traits at maturity. (**A, D**) Quantification of ear length at maturity in (**A**) 2019 and (**D**) 2020. (**B, E**) Quantification of ear diameter at maturity in (**B**) 2019 and (**E**) 2020. (**C, F**) Quantification of ear bald tip length at maturity in (**C**) 2019 and (**F**) 2020. (**G–I**) Correlation of (**G**) ear length, (**H**) ear diameter, and (**I**) bald tip length with kernel number. Lowercase letters indicate statistical significance groups at $p < 0.05$ (two-way analysis of variance with post-hoc least significant difference test). D1–D4 correspond to 75,000, 105,000, 120,000, and 135,000 plants/ha, respectively. Data are presented as the mean ± standard error from three biological replicates per group, each of which consisted of twenty samples.

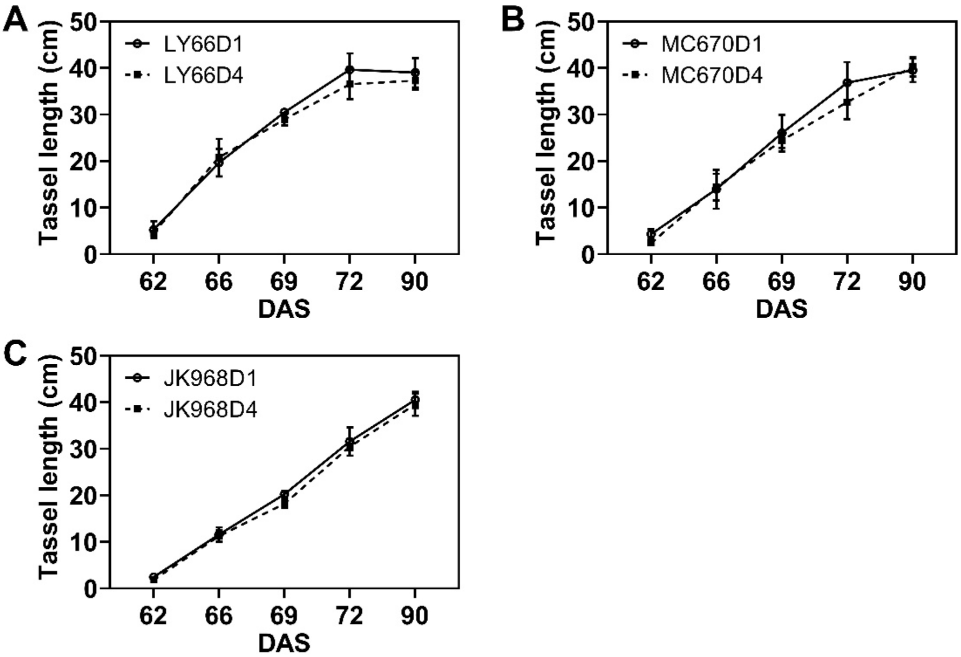

Supplemental Figure S9. Tassel lengths of three maize cultivars among plants grown at several planting densities.

(**A**–**C**) Tassel lengths of (**A**) LY66, (**B**) MC670, and (**C**) JK968 in 2020. DAS, days after sowing. Data are presented as the mean ± standard error from four biological replicates per group.

Supplementary Table S1. The average leaf length and average leaf width in each maize canopy layer among plants grown at several planting densities

Year	Cultivar	Plant density	Average leaf length (cm)				Average leaf width (cm)			
			Layer I	Layer II	Layer III	Layer IV	Layer I	Layer II	Layer III	Layer IV
2019	LY66	D1	64.31±5.60^{c}	94.93±4.65^{b}	88.69±3.66^{a}	54.31±4.75^{a}	7.25±0.48^{b}	9.50±0.33^{b}	9.95±0.29^{c}	7.89±0.36^{a}
		D2	70.88±2.15^{a}	101.02±4.40^{a}	86.28±4.34^{a}	48.38±4.36^{bc}	6.57±0.51^{c}	8.61±0.40^{c}	8.93±0.30^{d}	6.94±0.54^{b}
		D3	66.12±4.52^{bc}	101.04±2.75^{a}	86.92±4.76^{a}	45.61±3.98^{c}	5.83±0.20^{d}	8.35±0.37^{c}	8.53±0.39^{e}	6.33±0.56^{bc}
	MC670	D1	63.16±3.28^{c}	93.22±3.67^{b}	81.96±2.88^{b}	50.67±3.82^{ab}	7.72±0.52^{a}	10.71±0.59^{a}	11.50±0.44^{a}	7.64±0.81^{a}
		D2	63.84±1.93^{c}	93.67±1.36^{b}	77.22±2.75^{c}	45.26±3.18^{c}	7.17±0.29^{b}	9.64±0.28^{b}	10.38±0.34^{b}	6.88±0.41^{b}
		D3	68.67±4.12^{ab}	94.30±4.49^{b}	74.88±5.14^{c}	40.67±3.97^{d}	7.47±0.45^{ab}	9.81±0.58^{b}	10.14±0.46^{bc}	6.27±0.63^{c}
2020	LY66	D1	50.54±1.42^{d}	74.07±3.79^{d}	86.21±2.15^{bcd}	60.61±2.69^{a}	5.89±0.30^{de}	8.05±0.47^{f}	8.69±0.20^{f}	7.79±0.40^{abcd}
		D2	57.67±5.84^{c}	77.22±7.44^{cd}	85.50±2.56^{cd}	56.96±3.68^{abc}	6.19±0.34^{d}	7.77±0.16^{fg}	8.32±0.21^{fg}	7.22±0.55^{cde}
		D3	58.67±2.87^{bc}	78.27±5.24^{bcd}	84.90±3.16^{d}	53.55±2.52^{cd}	5.90±0.33^{de}	7.63±0.60^{fg}	8.04±0.17^{g}	6.81±0.36^{e}
		D4	58.38±0.89^{bc}	76.73±1.91^{cd}	84.70±2.29^{d}	54.42±1.61^{bcd}	5.77±0.30^{e}	7.52±0.26^{g}	7.97±0.19^{g}	7.00±0.50^{de}
	MC670	D1	55.32±0.66^{c}	80.21±1.93^{bc}	83.86±3.95^{d}	55.98±3.45^{bc}	7.24±0.17^{b}	9.49±0.16^{cd}	11.17±0.54^{ab}	8.33±0.63^{ab}
		D2	55.83±1.52^{c}	82.26±3.68^{b}	83.11±3.07^{d}	53.76±2.50^{bcd}	7.20±0.17^{b}	9.19±0.36^{de}	10.65±0.21^{cd}	7.72±0.59^{bcd}
		D3	56.73±0.36^{c}	82.07±1.88^{b}	82.82±0.43^{d}	53.06±2.65^{cd}	7.12±0.05^{bc}	8.92±0.16^{e}	10.20±0.17^{de}	7.10±0.57^{de}
		D4	59.23±1.65^{bc}	83.00±4.10^{b}	81.67±2.47^{d}	50.39±3.02^{d}	7.08±0.18^{bc}	8.83±0.39^{e}	10.14±0.31^{e}	7.17±0.41^{cde}
	JK968	D1	67.80±6.95^{a}	97.08±3.22^{a}	86.18±3.48^{bcd}	54.39±3.17^{bcd}	7.94±0.47^{a}	11.32±0.25^{a}	11.56±0.47^{a}	8.09±0.58^{ab}
		D2	56.78±1.67^{c}	96.05±5.51^{a}	91.03±6.51^{a}	57.94±5.99^{ab}	7.02±0.34^{bc}	10.23±0.46^{b}	11.37±0.78^{a}	8.54±1.16^{ab}
		D3	61.77±3.11^{b}	96.10±1.77^{a}	90.00±4.68^{ab}	55.99±3.19^{bc}	6.92±0.26^{bc}	9.92±0.43^{bc}	11.25±0.33^{ab}	8.58±0.65^{a}
		D4	56.46±2.56^{c}	95.75±4.21^{a}	89.44±3.81^{abc}	55.18±1.31^{bc}	6.76±0.44^{c}	9.86±0.60^{bc}	10.87±0.55^{bc}	7.95±0.60^{abc}

Data are presented as the mean ± standard error from three biological replicates parameter for each variety and year. Lowercase letters indicate statistical significance groups

at $p < 0.05$ (two-way analysis of variance). D1–D4 correspond to 75,000, 105,000, 120,000, and 135,000 plants/ha, respectively.

Supplementary Table S2. Days in the tasseling and silking stages among plants grown at several planting densities

Variety	Planting density	2019		2020		ASI (d)
		Tasseling (d)	Silking (d)	Tasseling (d)	Silking (d)	
LY66	D1	78	79	83	84	1-2
LY66	D2	78	81	84	87	2-3
LY66	D3	79	83	84	88	3-4
LY66	D4	-	-	84	87	3-4
MC670	D1	77	79	84	85	1-2
MC670	D2	78	81	85	87	2-3
MC670	D3	78	82	85	88	2-4
MC670	D4	-	-	85	88	3
JK968	D1	-	-	84	85	1
JK968	D2	-	-	86	88	1-2
JK968	D3	-	-	87	91	4
JK968	D4	-	-	87	93	5-6

ASI, anthesis–silking interval. Days to tasseling and silking were recorded as the number of days after sowing. D1–D4 correspond to 75,000, 105,000, 120,000, and 135,000 plants/ha, respectively.
